# Serious limitations of the current strategy to control Soil-Transmitted Helminths and added value of Ivermectin/Albendazole mass administration: A population-based observational study in Cameroon

**DOI:** 10.1371/journal.pntd.0008794

**Published:** 2020-11-03

**Authors:** Linda Djune-Yemeli, Hugues C. Nana-Djeunga, Cédric G. Lenou-Nanga, Cyrille Donfo-Azafack, André Domche, Floribert Fossuo-Thotchum, Yannick Niamsi-Emalio, Francine Ntoumi, Joseph Kamgno

**Affiliations:** 1 Centre for Research on Filariasis and other Tropical Diseases (CRFilMT), Yaoundé, Cameroon; 2 Molecular Diagnosis Research Group, Biotechnology Centre-University of Yaoundé I (BTC-UY-I), Yaoundé, Cameroon; 3 Parasitology and Ecology Laboratory, Department of Animal Biology and Physiology, Faculty of Sciences, University of Yaoundé I, Yaoundé, Cameroon; 4 Fondation Congolaise pour la Recherche Médicale, Brazzaville, Republic of the Congo; 5 Marien Ngouabi University, Brazzaville, Republic of the Congo; 6 Department of Public Health, Faculty of Medicine and Biomedical Sciences, University of Yaoundé I, Yaoundé, Cameroon; University of Pennsylvania, UNITED STATES

## Abstract

**Background:**

Soil-transmitted helminth (STH) infections remain a public health concern in sub-Saharan Africa. School-based mass drug administration (MDA) using the anthelminthic drug Mebendazole/Albendazole have succeeded in controlling morbidity associated to these diseases but failed to interrupt their transmission. In areas were filarial diseases are co-endemic, another anthelminthic drug (Ivermectin) is distributed to almost the entire population, following the community-directed treatment with ivermectin (CDTI) strategy. Since Ivermectin is a broad spectrum anthelmintic known to be effective against STH, we conducted cross-sectional surveys in two health districts with very contrasting histories of Ivermectin/Albendazole-based PC in order to investigate whether CDTI might have contributed in STH transmission interruption.

**Methodology:**

Cross-sectional surveys were conducted in two health districts with similar socio-environmental patterns but with very contrasting CDTI histories (Akonolinga health district where CDTI was yet to be implemented vs. Yabassi health district where CDTI has been ongoing for two decades). Stool samples were collected from all volunteers aged >2 years old and analyzed using the Kato-Katz technique. Infections by different STH species were compared between Akonolinga and Yabassi health districts to decipher the impact of Ivermectin/Albendazole-based MDA on STH transmission.

**Principal findings:**

A total of 610 and 584 participants aged 2–90 years old were enrolled in Akonolinga and Yabassi health districts, respectively. Two STH species (*Ascaris lumbricoides* and *Trichuris trichiura*) were found, with prevalence significantly higher in Akonolinga health district (43.3%; 95% CI: 38.1–46.6) compared to Yabassi health district (2.5%; 95% CI: 1.1–5.1) (chi-square: 90.8; df: 1; p < 0.001).

**Conclusion/significance:**

These findings (i) suggest that Mebendazole- or Albendazole-based MDA alone distributed only to at-risk populations might not be enough to eliminate STH, (ii) support the collateral impact of Ivermectin/Albendazole MDA on *A*. *lumbricoides* and *T*. *trichiura* infections, and (iii) suggest that Ivermectin/Albendazole-based PC could accelerate STH transmission interruption.

## Author summary

Decades of preventive chemotherapy (PC) using Mebendazole/Albendazole among school-aged children had not significantly decreased STH prevalence and intensity of infection, and Mathematical modelling demonstrated that interruption in STH transmission cannot be achieved unless MDA are broadened to an important proportion of the entire population. Concurrently, Ivermectin has been considered as a wonder drug because of (i) its applications in veterinary medicine, (ii) its tremendous impact on its initial indication in human medicine (river blindness), and (iii) its large spectrum of activity up to quite unsuspected effects on Human Immunodeficiency Virus (HIV). The efficacy of Ivermectin on STH has therefore been largely documented since the 2000s. Since Ivermectin/Albendazole-based MDA is administered to almost the entire population, we conducted cross-sectional surveys in two health districts with very contrasting histories of Ivermectin/Albendazole mass distributions to investigate whether Ivermectin/Albendazole-based PC might have interrupted STH transmission. Our findings confirm that Mebendazole- or Albendazole-based MDA alone and administered only to at-risk groups is not enough to eliminate STH and suggest that PC with Ivermectin (in combination to Albendazole) administered almost to the entire population would have contributed to STH transmission interruption. These observations are supportive of revision of WHO guidelines and recommendations to fight against STH.

## Introduction

Soil-transmitted helminth (STH) infection is caused by different species of parasitic nematode worms including the roundworm (*Ascaris lumbricoides*), the whipworm (*Trichuris trichiura*) and hookworms (*Necator americanus* and *Ancylostoma duodenale*). STH are widely distributed in tropical and subtropical countries where the soil is warm and moist [1]. Approximately 1.5 billion people are infected with STH worldwide, which is equivalent to about 24% of the world's population [2]. Globally, STH are responsible for the loss of 5.18 million Disability Adjusted Life Years (DALYs); with the highest burden in Southeast Asia (47%) and sub-Saharan Africa (SSA) (23%) [3]. Morbidity of STH is directly linked to worm burden and hence the greater the number of worms in an infected person, the greater the severity of the disease. The burden of STH is heavier in low- and middle-income countries, mainly because of poor sanitation and lack of adequate water supplies [4]. STH control strategies are currently focused on preventive chemotherapy (PC) with broad spectrum anthelminthic drugs, with the aim of alleviating morbidity through reductions in parasite burdens [5]. WHO recommends annual treatment in areas where the prevalence is between 20% and 50%, and a bi-annual treatment in areas with prevalence over 50% [6].

In Cameroon, more than 10 million persons suffer from intestinal worms with approximately 7.6 million children at risk of STH infection [4,7]. A strategic plan for STH control was developed in 2004, and since 2007, routine annual deworming of pre-school- and school-aged children with Mebendazole (MEB) has been implemented nationwide [8]. In areas were filarial parasites are also endemic, other anthelminthic drugs (Ivermectin [IVM] and Albendazole [ALB]) are distributed at community level through the community-directed treatment with IVM (CDTI) strategy.

IVM is a broad-spectrum anthelmintic which has entirely transformed strategies for the control and/or elimination of onchocerciasis and Lymphatic Filariasis (FL) since its registration for human health in 1987 [9]. Indeed, in addition to its efficacy against filarial nematode, IVM has exhibited significant effects on a wide range of endo- and ectoparasites (including STH, scabies, pediculosis, gnathostomiasis and myiasis) [10–14]. In addition, ALB, another benzimidazole drug which shown higher efficacy on *A*. *lumbricoides* and hookworm than MEB [15], is used in combination with IVM by LF elimination program [16]. Despite the separate efficacy of each of these drugs, it was demonstrated that they are more effective when used in combination. To assess whether IVM (in combination with ALB) mass administration may play a role in interrupting the transmission of STH, we compared prevalence and intensity of STH infections in two health districts in Cameroon with a very contracting history of IVM and/or IVM+ALB-based PC.

## Methods

### Ethics approval and consent to participate

An ethical clearance was obtained from the Faculty of Medicine and Biomedical Sciences Institutional review board (N°294/UY1/FMSB/VDRC/CSD) and administrative authorizations were granted by the Akonolinga and Yabassi District Medical Officers. Prior to the beginning of the surveys, the objectives and schedules of the study were explained to all the participants. Participation was entirely voluntary and any individual (or parent of a child) was free to opt out without fear of retaliation from their community leaders and program personnel. Formal written consent was obtained from participants and parent/guardian of minors prior enrollment.

### Study areas and populations

The present study was conducted in two health districts in Cameroon, exhibiting similar epidemiology of STH.

**Fig 1:**
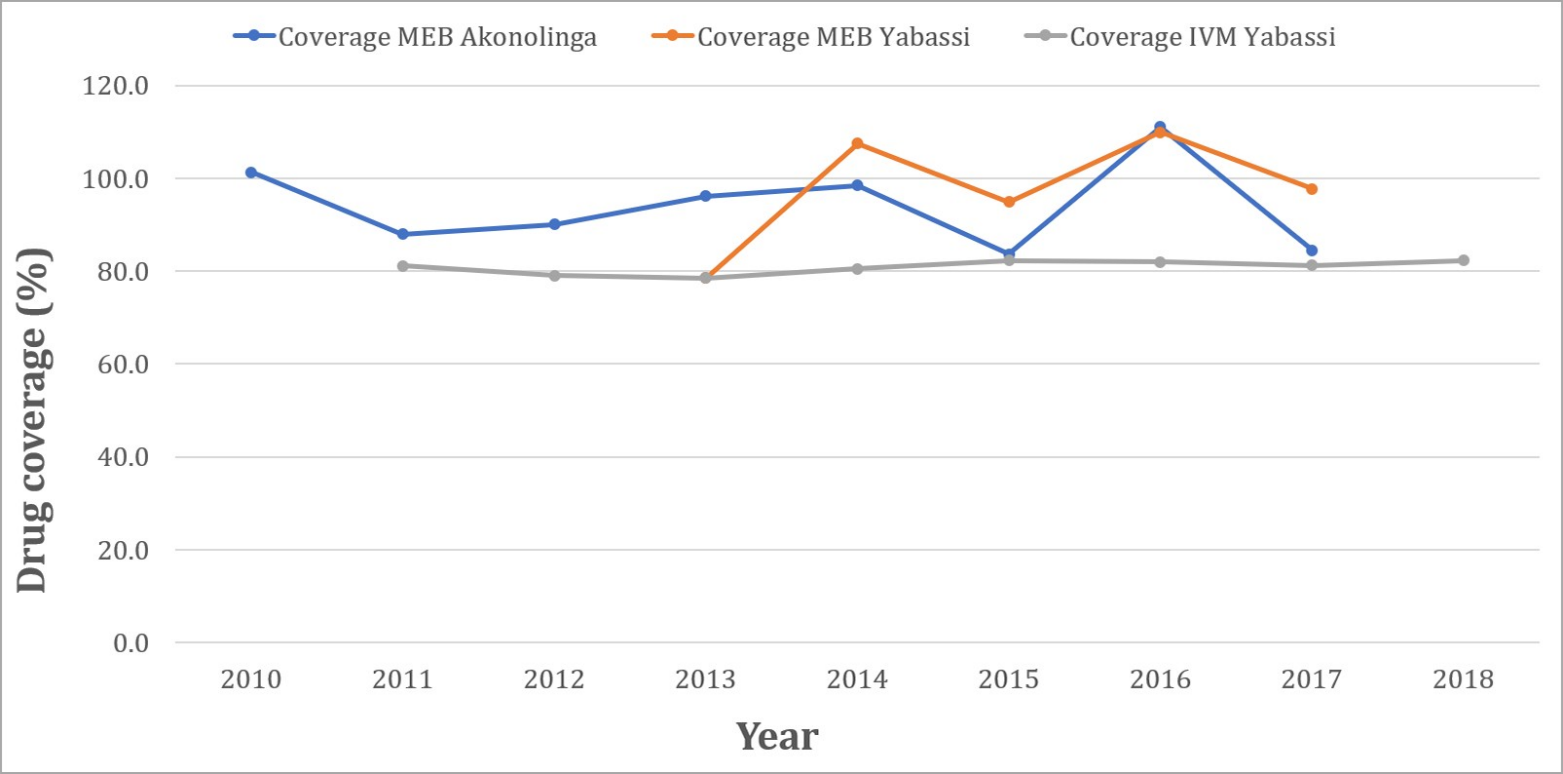
Reported therapeutic coverages in the Yabassi and Akonolinga health districts. Data were gathered on ESPEN website and/or provided by control programmes.

The Akonolinga health district is situated in the Nyong-et-Mfoumou Division (Centre Region, Cameroon), and belongs to a forested environment. Its relief has a plateau configuration, the highest point culminating at 877 m. The climate is of equatorial type divided into four seasons, with mean temperatures equal to 24.2°C and mean precipitations of 1,572 mm. The populations of this health district are typically rural, and their main activities are farming (coffee, cocoa, and food crops) and fishing [17]. Baseline data on STH in the Nyong-et-Mfoumou Division in 1985 revealed that *A*. *lumbricoides* (83.2%) and *T*. *trichiura* (98.2%) were highly endemic in this area [18,19]. A follow-up study conducted in the Akonolinga health district in 2015 reported prevalences of 18.0%, 43.7% and 7.5% for *A*. *lumbricoides*, *T*. *trichiura*, and *N*. *americanus*, respectively [20]. This health district was also known to be hypo-endemic for onchocerciasis and highly endemic for loiasis and as such, CDTI is yet to be implemented. Only MEB-based MDA is implemented in the framework of nationwide deworming campaigns since 2007, with very high drug coverage (>80%) among Pre-school and school-aged children (Fig 1).

The Yabassi health district is located in the Nkam Division (Littoral Region, Cameroon), at ~100 km north-east from Douala, the economic capital of the country. The relief is undulating, with valleys and plains, and the altitude varies from 10 to 800m. The vegetation is mainly dense humid forest. The climate is of equatorial type divided into two seasons, with mean temperatures equal to 22.5°C and mean precipitations of 1,364 mm. Because of the intense hydrographic network (Nkam, Dibamba, Makombé …) and the dense vegetation cover maintained by abundant precipitation, the relative humidity is high, favoring the development of the chrysops, vectors of the loiasis. Agriculture is the main activity, interesting at least 60% of inhabitants. According to a survey conducted in 1985–1987 on the distribution of STH in Cameroon, the Nkam division was highly endemic to STH, with prevalence of 61.4% for *A*. *lumbricoides*, 88.2% for *T*. *trichiura* and 23.2% for hookworms) [18,19]. Since onchocerciasis and lymphatic filariasis were also known to be highly endemic in this health district, the combination of IVM and ALB is distributed in the framework of large-scale community-based (through CDTI) treatment campaigns since 2000, with therapeutic coverages >80% (Fig 1). Since STH were also known to be moderately endemic in this health district, MEB is distributed, with very high drug coverage (>80%) (Fig 1), to Pre-school- and school-aged children as part of deworming organized since 2007. Both MDA campaigns (MEB-based and IVM+ALB-based) are not distributed simultaneously but staggered at different time periods of the year.

### Study design

Two cross-sectional surveys were conducted to compare prevalence and intensity of STH infections in two settings with similar socio-environmental patterns but contrasting history of IVM/ALB mass administration. To complement reported drug coverages (Fig 1) and take into account memory biases, a five-year compliance to MDA (either CDTI or MEB/ALB-based PC) was conducted prior to the assessment and comparison of prevalence and intensity of STH infection between the two settings. Considering previous STH prevalence in both sites (51.5% and 65.0% in the Akonolinga and Yabassi health district, respectively), type I error of 5% and a power of 80%, the minimal sample size in each group was 206.

### Sample collection and processing

A 60mL plastic screw-cap vial was provided to all the eligible participants for stool collection. These stool samples were analyzed using Kato-Katz technique, consisting in a single thick smear technique using a 41.7mg template [21]. The preparation was examined by qualified laboratory technicians, within one hour after slides’ preparation to ensure that all STH species potentially present in the preparation should be identified. All eggs found in the preparations were identified and counted using bright field microscopy (magnification x100 or x400) and the results expressed as eggs per gram of stool (epg).

### Statistical analysis

All relevant data were recorded into a Microsoft Excel spread sheet and subsequently exported to GraphPad Prism for statistical analysis. Prevalence or infection rates were expressed as percentage with 95% confidence interval; intensities of infection were expressed as means with standard deviations (SD). Chi-square test was used to compare prevalence or infection rates between health districts, genders and age groups, and Student t-test for independent samples was used to compare mean intensity of infection between different health districts, genders and age groups. Logistic or quasi-Poisson regression models were performed to control possible confounders and identify factors (health district, gender, age group) associated with the prevalence or intensity of STH infection. The threshold for significance was set at 5% for all statistical analyses.

## Results

A total of 1,194 individuals were enrolled in the present study among whom 610 in the Akonolinga health district and 584 in the Yabassi health district. The sex ratio was female-Biased, both in Akonolinga (0.87) and Yabassi (0.81) health districts. The enrollees were aged 2–90 years old, the median age being 23 years old (Inter-quartile range (IQR): 9–52) and 14 years old (IQR: 6–37) in the Yabassi and Akonolinga health districts, respectively.

### Adherence to mass drug administration

**Fig 2:**
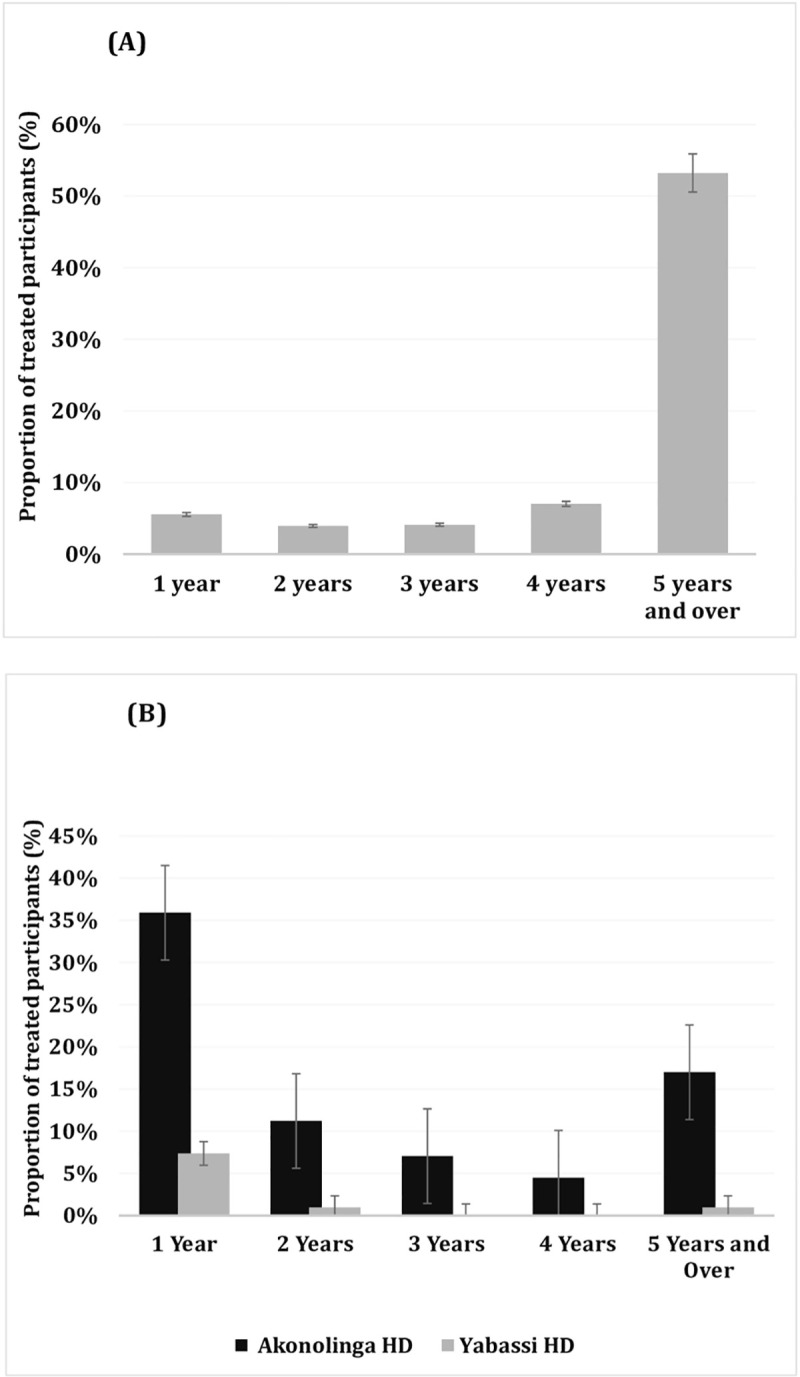
Adherence to IVM/ALB and MEB mass treatments. (A) Represents the proportion of participant treated with IVM and/or ALB in the Yabassi health district; (B) Shows the proportion of Pre- and School aged children treated with MEB in the Yabassi and Akonolinga health districts.

In the Yabassi health district where CDTI is ongoing, 73.9% (95% CI: 70.6–77.7) of participants reported having taken IVM and ALB at least once, and 53.2% (95% CI: 49.2–57.3) declared having taken IVM+ALB during the last five years (Fig 2A). Regarding compliance to MEB-based PC, of the 312 preschool- and school-aged children recruited in the Akonolinga health district (area without CDTI), 236 (75.6%; 95% CI: 70.6%-80.1%) reported to have taken MEB at least once. In the Yabassi health district, 61.7% (95% CI: 47.4%-74.2%) declared to adhere to MEB-based MDA (Fig 2B). An increasing trend was observed in the number of IVM+ALB-based PC, while a decreasing trend was observed in the number of MEB-based MDA, as per interviewees’ declarations.

### STH prevalence

A total of 514 and 284 individuals provided stool samples in the Akonolinga and Yabassi health districts, respectively. The analysis of these stool samples revealed the presence of two STH species, *A*. *lumbricoides* and *T*. *trichiura*.

STH infections were similar between females and males, either by *A*. *lumbricoides* (Chi-square: 0.9; df: 1; p = 0.34) or by *T*. *trichiura* (Chi-square: 0.24; df: 1; p = 0.6) (Table 1). Regarding age groups, school-aged children (6–14 years old)—target of the national control program—were significantly more infected either by *T*. *trichiura* or by *A*. *lumbricoides* than their older counterparts (p<0.006) (Table 1).

**Table 1:** STH infection rates in the Akonolinga and Yabassi health districts according to gender and age groups.

Variable	Yabassi health district			Akonolinga health district		
	N examined	% infected with *A*. *lumbricoides* (95% CI)	% infected with *T*. *trichiura* (95% CI)	N examined	% infected with *A*. *lumbricoides* (95% CI)	% infected with *T*. *trichiura* (95% CI)
***Gender***						
Males	137	0.0% (0.0–2.7)	2.2% (0.7–6.2)	245	27.8% (22.2–33.8)	26.9% (21.5–33.0)
Females	147	2.0% (0.7–5.8)	0.7% (0.1–3.7)	269	33.1% (27.4–39.1)	24.5% (19.5–30.1)
***Age groups***						
2–5	30	0.0% (0.0–11.3)	6.7% (1.8–21.3)	117	11.1% (6.6–18.1)	27.3% (20.1–36.0)
6–14	84	1.2% (0.2–6.4)	1.2% (0.2–6.4)	157	52.9% (45.1–60.5)	35.7% (28.6–43.4)
15-Over	170	1.2% (0.2–4.6)	0.6% (0.1–3.3)	240	25.4% (20.3–31.3)	17.5% (13.2–22.8)
**Overall**	**284**	**1**.**1 (0**.**2–3**.**2)**	**1**.**5 (0**.**4–3**.**7)**	**514**	**30.5 (26.6–34.7)**	**25.7 (22.0–29.7)**

The prevalence of *A*. *lumbricoides* was 30.5% (95% CI: 26.6–34.7) in the Akonolinga health district and 1.1% (95% CI: 0.2–3.2) in the Yabassi health district, the difference being significant (Chi-square: 72.1; df: 1; p< 0.0001) (Table 1). Similarly, the prevalence of *T*. *trichiura* was significantly higher in the Akonolinga health district (25.7%; 95% CI: 22.0–29.7) than in the Yabassi health district and (1.5%; 95% CI: 0.4–3.7) (Chi-square: 57.9; df: 1; p<0.0001) (Table 1).

Multivariate logistic regression confirmed that the increased in STH prevalence was associated with the Akonolinga health district (p<0.0001) and individuals aged 6–14 years old (p<0.0001), but not with the gender (S1 Table).

### Intensities of infection

Table 2 shows the distribution of the intensity of STH infections according to gender and age groups. The intensity of infection, whatever the STH species, was similar between age groups. Regarding gender, although no significant difference was observed in the intensity of infection by *A*. *lumbricoides* between males and females, the mean number of *T*. *trichiura* eggs per gram of stool was significantly higher among females than males (p = 0.04).

**Table 2:** Intensity of STH infections in Akonolinga and Yabassi health districts according to gender and age groups.

Variable	Yabassi health district			Akonolinga health district		
	N examined	Mean *A*. *lumbricoides* epg (SD)	Mean *T*. *trichiura* epg (SD)	N examined	Mean *A*. *lumbricoides* epg (SD)	Mean *T*. *trichiura* epg (SD)
***Gender***						
Males	137	0.0 (0.0)	1.05 (7.6)	245	2513.7 (9316.6)	31.2 (98.0)
Females	147	0.65 (4.8)	0.33 (4.0)	269	2187.9 (7934.0)	55.8 (228.9)
***Age groups***						
2–5	30	0.0 (0.0)	2.4 (9.6)	117	1555.1 (8244.1)	47.2 (173.3)
6–14	84	0.29 (2.6)	0.9 (7.9)	157	3823.2 (9867.7)	56.3 (197.6)
15-Over	170	0.42 (4.1)	0.30 (3.7)	240	1759.3 (7786.5)	34.6 (169.2)
**Overall**	**284**	**0.3 (3.5)**	**0.7 (6.0)**	**514**	**2343.2 (8613.8)**	**44.1 (179.1)**

**Fig 3:**
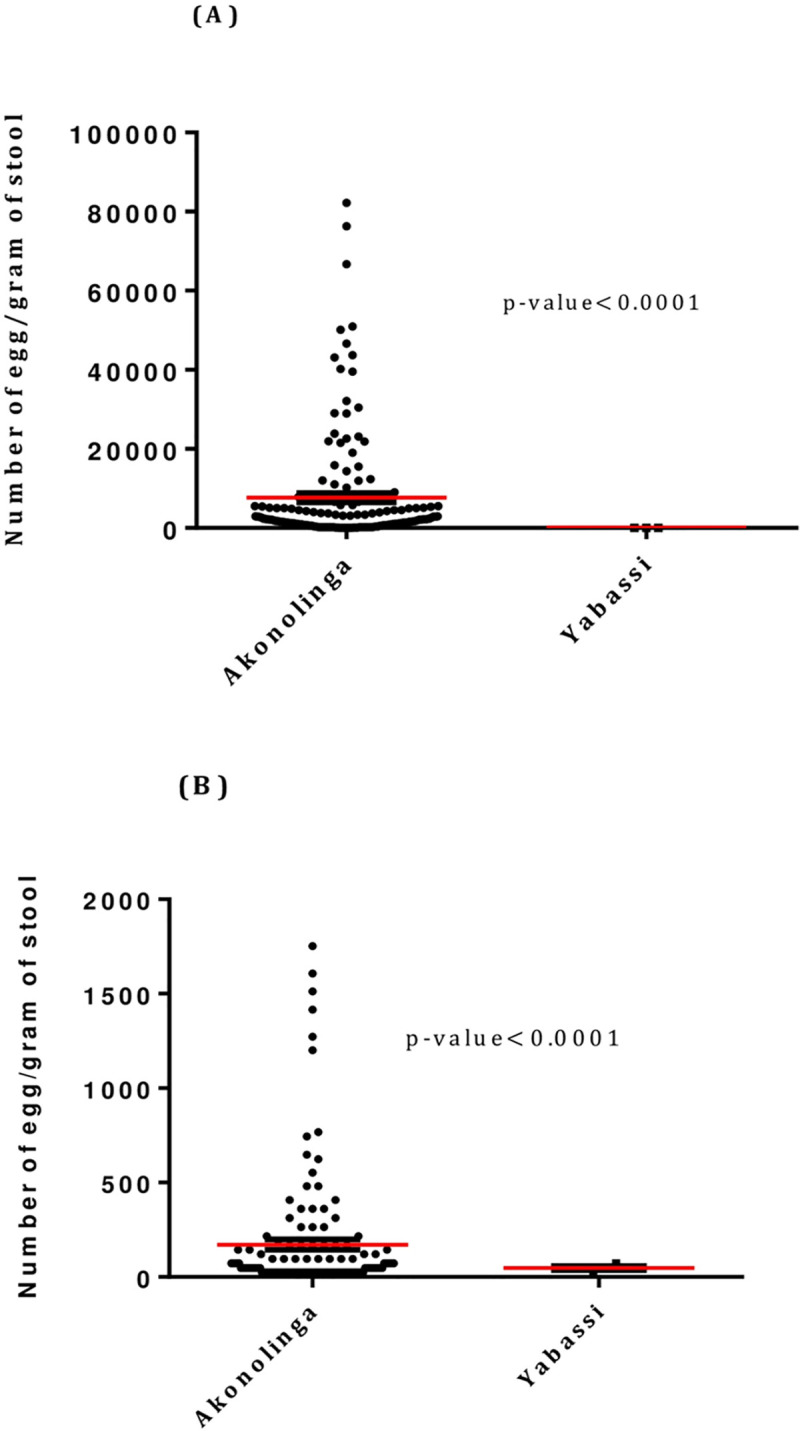
Intensities of STH infections in the Akonolinga (dark bars) and Yabassi (grey bars) health districts. Each black dot represents the number of eggs harbored by a single participant, either for *A*. *lumbricoides* (A) or for *T*. *trichiura* (B). The red line represents the mean number of eggs in each of the health districts.

The intensity of infection by *A*. *lumbricoides* was significantly higher (p<0.0001) in the Akonolinga health district (Mean: 2343.2epg; SD: 8613.8) than in the in the Yabassi health district (Mean: 0.3 epg; SD: 3.5) (Table 2; Fig 3A). Similarly, the mean number of *T*. *trichiura* eggs found per gram of stool was significantly higher (p<0.0001) in the Akonolinga health district (Mean: 44.1; SD: 179.1) compared to the Yabassi health district (Mean: 0.7; SD: 6.0) (Table 2; Fig 3B).

The quasi-Poisson regression model revealed that the intensity of STH infection, especially *T*. *trichiura*, was correlated only with health district (p = 0.009), the other co-variates (age group, gender) being not significantly linked to intensity of infection (S2 Table).

## Discussion

STH infections are endemic in Cameroon since several decades [18,19]. In 2007, MEB-based PC was undertaken in the 189 endemic health districts by the National Program for Schistosomiasis and Intestinal Helminthiasis Control (PNLSHI) to reduce burden and morbidity associated to STH infections [8]. Despite these efforts, STH infections remain highly prevalent and constitute a major public health concern in Cameroon, suggesting that control efforts, notably PC, are not enough to interrupt transmission of STH [7,8]. Indeed, the prevalence and intensity of infection observed in the Akonolinga health district in the present study were quite high, and even higher compared to their level in 2015 [20]. Importantly, this persistence of STH infection was observed in a context where the compliance to MEB-based PC among preschool- and school-aged children was high (>75%) in this health district. Although compliance to treatment was only based on the declarations of enrollees (or their parents/legal guardians), this observation suggests that adults who are not included in deworming campaigns might contribute in the persistence of STH in the Akonolinga health district as previously evoked [20].

Mathematical models have recently demonstrated that if STH transmission is to be interrupted by PC alone, treatment levels and frequency must be much higher, and the breadth of coverage across age classes broader than is typically the current practice [22]. In the Yabassi health district where CDTI has been ongoing since ~20 years at the onset of this study, the prevalence and intensity of STH are quite low (1.1% for ascariasis and 1.5% for trichuriasis). Indeed, the control of onchocerciasis using IVM (CDTI) was initiated in the 1990’s in the Yabassi health district, and ALB was introduced, in combination with IVM, since 2011 for the treatment of lymphatic filariasis. These PC are organized in the framework of the CDTI and involved individuals aged 5 years and over. The joint anthelmintic efficacy of IVM and ALB on STH, together with the broader treatment coverage and the long-term MDA history, might explain the important impact (to almost transmission interruption) in the Yabassi health district. It is worth to mention that a survey conducted in 1985 (that is prior MDA against both onchocerciasis/lymphatic filariasis and STH) in the Nkam Division to which the Yabassi health district belongs revealed prevalence of 61.4% and 88.2% for *A*. *lumbricoides* and *T*. *trichiura*, respectively, suggesting that STH were likely highly endemic in the Yabassi health district [18].

The efficacy of IVM against STH infections has already been demonstrated, and the impact of several years of mass IVM administration for the control of onchocerciasis on the dynamics of STH infections has been reported in several countries [11–13]. However, there are evidence of an increase therapeutic outcomes when IVM is co-administered with either MEB or ALB [23–25]. A STH transmission mathematical model indeed demonstrated that IVM co-administration with either MEB or ALB greatly increased the feasibility of and timeframe for breaking STH transmission [26]. Besides this, ALB is highly efficacious on *A*. *lumbricoides* and hookworms as compared to MEB [15]. Therefore, considering that MEB is the only drug distributed in the Akonolinga health district, together with the staggered distribution of MEB and IVM+ALB in the Yabassi health district at different time periods of the year, it is likely that the impact on the figures/trends observed in the Yabassi health district results from cumulative impact of both PC (MEB and IVM+ALB) distributed twice a year at different time periods. Indeed, it was recently demonstrated that semiannual MDA with ALB alone can exhibit a major impact on STH infections [27]. It is worth to mention that single dose IVM exhibits low/poor efficacy against hookworms, especially *Necator americanus* [28]. However, data documenting the long-term impact of IVM on this parasite species in the general population are scanty. To the best of our knowledge, this study is the first ever population-based study reporting the long-term (two decades) effect of IVM on STH endemicity in the general population (not only among school-aged children). Therefore, even though single dose IVM might be poorly effective on hookworms (especially *N*. *americanus*), repeated doses of IVM might have exhibited a significant impact on the latter.

The present study suggests that CDTI (combined with ALB) has indeed led to an important impact on dynamics of STH infections, but more importantly demonstrated that this strategy can help interrupting transmission of these highly morbid diseases. This study also confirms hypotheses raised by mathematical modelling on the fact that MDA should covered an important proportion of the entire population if one is expecting transmission interruption. Should the guidelines and strategies designed to fight against STH be revised, this study is informative and may be used for advocacy.

## Limitations

The main limitation of this study is the availability of community-based baseline data, especially in the Yabassi health district, that will ascertain that IVM/ALB-based PCT would be the main driver in transmission interruption of STH infections. Although no specific baseline data exist for this setting, prior MDA implementation prevalence have been documented in the Nkam Division to which the Yabassi health district belongs and is the capital, confirming that this health district was likely highly endemic to STH infections prior implementation of IVM/ALB-based PCT. Further studies in areas where baseline data exist is therefore highly needed to confirm the findings of the present study.

Also, only one slide was collected to establish infection with STH though additional slides or more sensitive diagnostic tools would have been more informative, especially when the prevalence and intensities of infections are low. First, the Kato-Katz technique was used, both in Yabassi and Akonolinga health districts, for comparison purpose since this diagnostic tool was used in previous/baseline studies. Furthermore, it has been demonstrated that variations between slides made from the same stool specimen are low [29], especially when stool samples are well homogenized before slides’ preparations.

## Conclusion

The present study (i) confirms that MEB- or ALB-based MDA alone distributed to at-risk populations might not be enough to eliminate STH, (ii) support the collateral impact of IVM/ALB MDA on prevalence and intensity of *A*. *lumbricoides* and *T*. *trichiura* infections, and (iii) suggest that IVM-based PC could accelerate STH transmission interruption. Further investigations on MEB/ALB-based PC in the entire population should be assessed to challenge the conclusions of mathematical modelling.

## Supporting information

S1 Table Association between prevalence of STH infection and health districts, genders, and age groups. (DOCX)

S2 Table Association between intensity of STH infection and health districts, genders, and age groups. (DOCX)
