# Supplementary material for: Serious limitations of the current strategy to control Soil-Transmitted Helminths and added value of Ivermectin/Albendazole mass administration: A population-based observational study in Cameroon
Source: PLoS Negl Trop Dis. 2020 Nov 3;14(11):e0008794. doi: 10.1371/journal.pntd.0008794 (PMC7665818; doi:10.1371/journal.pntd.0008794)
Supplement: S1 Table — (DOCX) [file pntd.0008794.s001.docx]

**S1 Table.** Association between prevalence of STH infection and health districts, genders, and age groups

| **STH species** | ***Ascaris lumbricoides*** | | ***Trichuris trichiura*** | |
| --- | --- | --- | --- | --- |
|  | **OR (95% CI)** | **AOR (95% CI)** | **OR (95% CI)** | **AOR (95% CI)** |
| Intercep | - | 0.5 (0.4 – 0.6) ^***^ | - | 0.32 (0.2 – 0.4) ^***^ |
| **Health District** |  |  |  |  |
| Akonolinga (ref) | 1 | 1 | 1 | 1 |
| Yabassi | 0.02 (0.0 – 0.1) ^***^ | 0.02 (0.0 – 0.1) ^***^ | 0.04 (0.01 – 0.1) ^***^ | 0.04 (0.01 – 0.09) ^***^ |
| **Gender** |  |  |  |  |
| Female (ref) | 1 | 1 | 1 | 1 |
| Male | 0.76 (0.5 – 1.1) ^ns^ | 0.74 (0.5 – 1.1) ^ns^ | 1.15 (0.8 – 1.7) ^ns^ | 1.2 (0.8 – 1.7) ^***^ |
| **Age group** |  |  |  |  |
| 2 – 5 (ref) | 1 |  | 1 |  |
| 6 – 14 | 5.51 (3.0 – 10.8) ^***^ |  | 1.03 (0.6 – 1.7) ^ns^ |  |
| 15 – Over | 1.87 (1.0 – 3.7) ^*^ |  | 0.41 (0.2 – 0.7) ^***^ |  |

*OR: crude odds ratio; AOR: adjusted odds ratio; CI: confidence interval; ref: reference category; *** p<0.0001; ** p<0.001; * p<0.05; ns: not significant*
