# Supplementary material for: Serious limitations of the current strategy to control Soil-Transmitted Helminths and added value of Ivermectin/Albendazole mass administration: A population-based observational study in Cameroon
Source: PLoS Negl Trop Dis. 2020 Nov 3;14(11):e0008794. doi: 10.1371/journal.pntd.0008794 (PMC7665818; doi:10.1371/journal.pntd.0008794)
Supplement: S2 Table — (DOCX) [file pntd.0008794.s002.docx]

**S2 Table.** Association between intensity of STH infection and health districts, genders, and age groups

| **Variable** | **Logarithm of *Ascaris lumbricoides*** | | | **Logarithm of *Trichuris trichiura*** | | |
| --- | --- | --- | --- | --- | --- | --- |
|  | **Coefficient** | **Standard error** | ***p*-value** | **Coefficient** | **Standard error** | ***p*-value** |
| **Health District** |  |  |  |  |  |  |
| Akonolinga (ref) | 1 |  |  | 1 |  |  |
| Yabassi | -8.84 | 14.54 | 0.543 | -4.18 | 1.60 | 0.009 |
| **Gender** |  |  |  |  |  |  |
| Female (ref) | 1 |  |  | 1 |  |  |
| Male | 0.13 | 0.33 | 0.691 | -0.57 | 0.355 | 0.108 |
| **Age** |  |  |  |  |  |  |
| 2 – 5 (ref) | 1 |  |  | 1 |  |  |
| 6 – 14 | 0.70 | 0.49 | 0.158 | -0.02 | 0.47 | 0.951 |
| 15 – Over | -0.18 | 0.51 | 0.723 | -0.62 | 0.47 | 0.190 |

*ref : reference category*
